# Maternal antibody interference contributes to reduced rotavirus vaccine efficacy in developing countries

**DOI:** 10.1371/journal.ppat.1009010

**Published:** 2020-11-19

**Authors:** Claire E. Otero, Stephanie N. Langel, Maria Blasi, Sallie R. Permar

**Affiliations:** 1 Duke Human Vaccine Institute, Duke University Medical Center, Durham, North Carolina, United States of America; 2 Department of Pathology, Duke University School of Medicine, Durham, North Carolina, United States of America; 3 Department of Pediatrics, Duke University Medical Center, Durham, North Carolina, United States of America; 4 Department of Medicine, Duke University Medical Center, Durham, North Carolina, United States of America; University of Wisconsin Madison, UNITED STATES

## Abstract

Rotavirus (RV) vaccine efficacy is significantly reduced in lower- and middle-income countries (LMICs) compared to high-income countries. This review summarizes current research into the mechanisms behind this phenomenon, with a particular focus on the evidence that maternal antibody (matAb) interference is a contributing factor to this disparity. All RV vaccines currently in use are orally administered, live-attenuated virus vaccines that replicate in the infant gut, which leaves their efficacy potentially impacted by both placentally transferred immunoglobulin G (IgG) and mucosal IgA Abs conferred via breast milk. Observational studies of cohorts in LMICs demonstrated an inverse correlation between matAb titers, both in serum and breast milk, and infant responses to RV vaccination. However, a causal link between maternal humoral immunity and reduced RV vaccine efficacy in infants has yet to be definitively established, partially due to limitations in current animal models of RV disease. The characteristics of Abs mediating interference and the mechanism(s) involved have yet to be determined, and these may differ from mechanisms of matAb interference for parenterally administered vaccines due to the contribution of mucosal immunity conferred via breast milk. Increased vaccine doses and later age of vaccine administration have been strategies applied to overcome matAb interference, but these approaches are difficult to safely implement in the setting of RV vaccination in LMICs. Ultimately, the development of relevant animal models of matAb interference is needed to determine what alternative approaches or vaccine designs can safely and effectively overcome matAb interference of infant RV vaccination.

## Rotavirus vaccine efficacy is reduced in lower- and middle-income countries (LMICs)

Despite the development of effective vaccines, which have reduced rotavirus (RV)-related morbidity and mortality by 67% [1], RV is still one of the most common causes of diarrheal disease in childhood [1,2]. There are currently 4 vaccines endorsed by the World Health Organization (WHO) to prevent RV-induced gastroenteritis: Rotarix, Rotateq, Rotavac, and RotaSiil, but only Rotarix and Rotateq are widely used globally [3]. These vaccines are orally administered, live-attenuated formulations, each containing different human and/or bovine serotypes of RV (Table 1). In first-world countries, RV vaccines are highly efficacious (80% to 90%), but in LMICs, efficacy plummets to 40% to 60% [4,5]. Due to this disparity in vaccine efficacy, RV infections still cause significant morbidity and mortality in LMICs [2].

**Table 1:** Current RV vaccines.

Vaccine	Developer	WHO prequalified	Composition	matAb interference reported?
RotaTeq	Merck (United States)	2008	Pentavalent human–bovine reassortant G1–G4 and P[6] [7]	Yes [8]
Rotarix	GlaxoSmithKline (Belgium)	2009	Monovalent human G1P[6] [6]	Yes [9]
Rotavac	Bharat Biotech (India)	2018	Monovalent human–bovine reassortant G9P[10] [3,11]	Yes [10]
Rotasiil	Serum Institute of India (India)	2018	Thermostable pentavalent human-bovine reassortant G1, G2, G3, G4, and G9 [12–14]	No^a^

^a^No references indicating matAb does or does not interfere.

RV, rotavirus; WHO, World Health Organization.

Several reasons for low RV vaccine efficacy in LMICs have been proposed, including higher RV exposure, greater diversity of RV G and P serotypes, malnutrition, microbiome composition, maturation stage of the immune system, reduced vaccine replication due to other enteric pathogens, coadministration of the oral polio virus vaccine, different expression of histo-blood group antigens, skewed T helper 1 (Th1)/T helper 2 (Th2) balance and antibody response to vaccination, and higher incidence of maternal antibody (matAb) interference [15–18]. While it is likely that multiple factors contribute to the reduced RV vaccine efficacy observed in LMICs, matAb interference is likely a major contributor due to greater RV exposure, leading to greater maternal immunity, and higher rates and longer duration of breastfeeding in LMICs [19–21]. This review focuses on current evidence supporting matAb interference as a contributor, remaining questions, and proposed modifications to increase the efficacy of current vaccine regimens.

## Evidence supports matAb interference as a mechanism of reduced RV vaccine efficacy

MatAbs are transferred to the infant via 2 distinct routes: (1) placental transfer of immunoglobulin G (IgG) into infant circulation; and (2) breast milk transfer of primarily IgA into the infant gastrointestinal tract [22,23]. Most studies investigating the role of matAb interference focus on placentally transferred IgG [24]. However, evidence from both population-level observational and animal modeling studies suggest that breast milk–derived matAb also interferes with RV vaccine efficacy [10,25,26].

Rotavac is a recently developed RV vaccine derived from a naturally attenuated and reassorted neonatal RV strain (116E). A clinical trial of this vaccine in Indian infants identified a significant inverse relationship between RV-specific maternal IgG and infant Rotavac vaccine responses. However, matAb inhibition was overcome by increasing the vaccine dose [10]. While the Rotavac trial did not investigate breast milk Abs as a contributor to matAb interference, modeling of RV infection using the murine RV strain Epizoonotic Diarrhea of Infant Mice (EDIM) showed that seropositive BALB/c dams conferred Abs to their pups, primarily through breastfeeding, which impaired pups’ immune responses to live RV inoculation [26]. These findings concur with studies of human cohorts in developing countries, such as Zambia and Vietnam, where babies of mothers with higher titers of anti-RV Abs in breast milk tend to have reduced responses to RV vaccines [9,20]. These observational studies demonstrate a consistent association between maternal humoral immunity, including both serum IgG and mucosal IgA, and infant responses to RV vaccines.

## Establishing a causal link between matAb interference and low RV vaccine efficacy in LMICs and defining mechanisms

While observational studies in animal and human populations have established a link between maternal immunity and infant vaccine efficacy, mechanistic studies demonstrating that matAb interference causes a reduction in RV vaccine efficacy are still needed. Previous studies have demonstrated an inverse correlation between serum and breast milk matAb titers and infant responses [9,27], but these studies do not isolate this effect to matAb alone. Further, there are several RV G/P serotypes in circulation [28], and maternal exposure to certain serotypes may impact the degree of matAb interference observed, depending on the level of cross-reactivity of matAbs between wild-type RV strains and attenuated vaccine viruses. There are many other potential immune factors conferred from mother to child that may inhibit infant vaccine responses. One study of Zambian children found that lactadherin, an antiviral glycoprotein present in breast milk, negatively associated with infant seroconversion after vaccination with Rotarix [29]. Additionally, genetic host factors, such as expression of histo-blood group antigens, a cellular receptor for RV, may also influence RV vaccine efficacy and susceptibility to disease [30–33]. Thus, studies in which matAb can be isolated as a variable are needed to establish a causal link to reduced RV vaccine efficacy. A major impediment to such studies is the difficulty in modeling human RV infection in animal models due to the limited host range of RVs [34].

**Fig 1:**
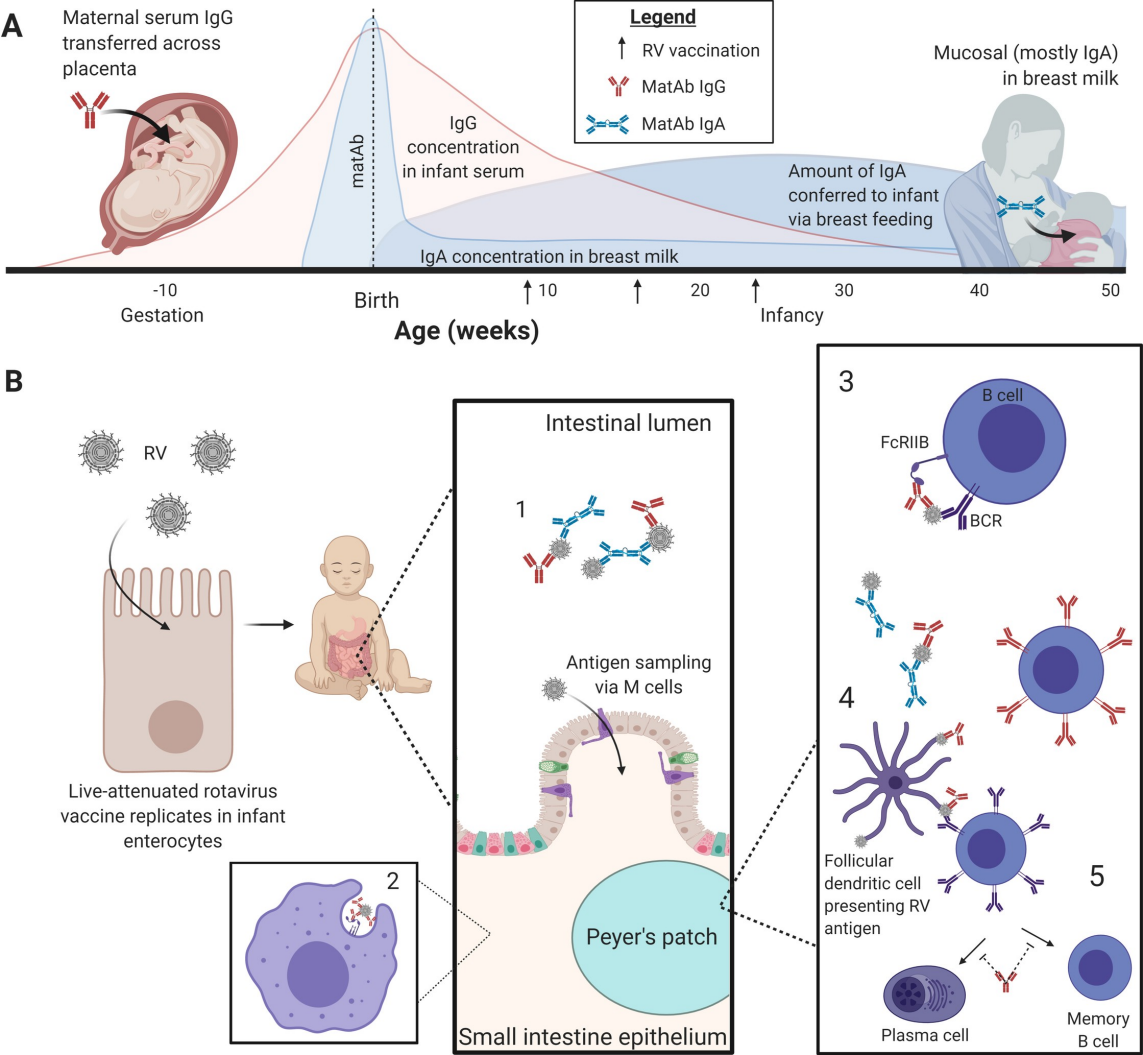
matAb interference to infant RV vaccines [41]. (A) Placentally transferred IgG (red curve) begins reaching the infant as early as 8 weeks of gestation and peaks at term, approximately 40 weeks [22,42]. Maternally derived IgG wanes in the infant over 12 months after birth [24]. Breast-fed infants receive Abs, primarily IgA, through breast milk, which peaks in colostrum at a concentration of approximately 12 mg/mL and maintains approximately 1 mg/mL in mature milk (light blue curve) [23]. However, due to the volume of milk consumed by the infant, the absolute amount of matAb transferred via breast milk increases over time until the child can start getting energy from other kinds of food (dark blue curve) [43]. RV vaccination typically occurs in 2 to 3 doses when the infant is 2 to 6 months old, as indicated by the black arrows [44]. In breast-fed infants, both types of matAb are present at the time of RV vaccination. (B) RV vaccines are orally administered live-attenuated viruses, which rely on replication in infant enterocytes to elicit a robust immune response. Microfold (M) cells sample antigens from the gut lumen and present them to antigen-presenting cells, which stimulate the adaptive immune response in Peyer’s patches [45]. In the presence of matAbs, several mechanisms have been proposed for reduction of infant immune responses to RV vaccination, including (1) inhibition of vaccine virus replication in enterocytes by matAb neutralization; (2) removal of vaccine antigen by antibody-mediated phagocytosis; (3) inhibition of infant B cell activation by cross-linking BCRs with inhibitory FcγRIIB; (4) epitope masking, which inhibits infant Ab responses by hiding recognizable antigens from infant B cells, which may also shift B cell responses toward nonimmunodominant epitopes; and (5) impacting downstream differentiation of B cells into plasma cells or memory B cells [24,35]. Ab, antibody; BCR, B cell receptor; FcγRIIB, Fcγ receptor IIB; IgA, immunoglobulin A; IgG, immunoglobulin G; matAb, maternal antibody; RV, rotavirus.

Several mechanisms have been proposed for IgG-mediated matAb interference against different viruses, including neutralization of live-attenuated vaccines, epitope masking, cross-linking of B cell receptors (BCRs) and inhibitory Fcγ receptor IIB (FcγRIIB), vaccine antigen removal via antibody-mediated phagocytosis, and downstream inhibition of B cell differentiation into plasma or memory B cells (Fig 1) [24,35]. A study of maternal IgG-mediated interference to measles live-attenuated vaccination in the cotton rat model demonstrated that nonneutralizing monoclonal Abs mediated interference, while neutralizing monoclonal Abs did not [36]. This study also indicated that the fragment crystallizable (Fc) region is necessary to inhibit Ab responses to vaccination and that this inhibition is due to interaction with FcγRIIB [36]. However, studies in mice utilizing sheep red blood cells as a model antigen have supported epitope masking as a mechanism mediating this inhibition of B cell responses [37–39]. Notably, one study demonstrated that interference occurs in FcγR-deficient mice, demonstrating that BCR–FcγRIIB is not the sole mechanism of B cell inhibition in the presence of preexisting Ab [39]. Interestingly, RV Abs targeting the middle capsid layer (VP6), which have traditionally been considered nonneutralizing, are capable of intracellular neutralization, suggesting that the impact of such maternal Abs on neonatal vaccine efficacy may not be limited to Fc-mediated “nonneutralizing” effector functions [40]. In another recent study using influenza hemagglutinin as a model antigen, researchers found that matAbs do not impact germinal center formation but modulate which antigens are targeted by infant B cells and, in a dose-dependent manner, inhibit B cell differentiation of plasma and memory B cells through an undefined mechanism [35]. Together, these results suggest that multiple mechanisms may contribute to matAb-mediated inhibition of infant vaccine responses, possibly in an antigen-dependent manner.

RV vaccines are orally administered, so they may also be affected by Abs at the intestinal mucosa, primarily IgA delivered to the infant via breast milk. Notably, IgA-mediated interference may not follow the same mechanism(s) as IgG-mediated interference due to differences in Fc characteristics. Additionally, it is noteworthy that while breast milk contains mostly IgA Abs, breast milk IgG Abs are present and can be transported to the lamina propria and into circulation [46,47]. However, studies in multiple LMIC populations have shown that abstaining from breastfeeding for a period before and after RV vaccination does not change seroconversion rates [48–51]. The ineffectiveness of breastfeeding timing on RV vaccination may indicate that circulating, rather than breast milk, maternal IgG is the primary mediator of the interference. Further and more in-depth evaluation of Ab characteristics and the relative contribution of serum IgG and breast milk IgA would be informative for design and evaluation of strategies to overcome matAb interference.

## Potential solutions for matAb interference to RV vaccines

Several strategies can help circumvent matAb interference, but each comes with its own risks. RV vaccination is associated with a slightly increased risk of intussusception, which is generally outweighed by the immense benefit of reduction in morbidity and mortality, but must be considered when evaluating alternative vaccination strategies [52]. For example, while increasing the vaccine antigen dose may overcome matAb interference [10], a matAb-exceeding dose could also lead to pathology due to excessive replication of live vaccine virus or an improper immune response. This approach was previously tested using a live-attenuated measles vaccine, which induced some protection in the presence of maternal IgG but also resulted in increased infant mortality, especially in girls, who tended to have less maternal IgG compared to boys [24,53–55]. Serology testing to quantitate preexisting Ab before vaccination is not feasible in LMICs, so it would not be possible to adjust the antigen dose based on matAb level, which would be an ideal compromise to improve the safety of this approach.

Another alternative strategy is changing the timing of vaccination to wait until matAb levels in the infant wane. The measles vaccine follows this strategy as administration after 9 months of age demonstrated reduction of matAb interference [56]. However, later vaccination can also pose a significant risk because it leaves the infant more vulnerable during the period before vaccination when matAb levels are low, which is an important consideration in LMICs with greater RV exposure [19]. However, passive immunotherapy of a breast milk–targeted antibody delivered to the mother may keep mucosal matAb at a protective level until vaccination at a later age. Thus, additional studies are needed to determine the age when RV-specific matAbs have waned enough to achieve successful vaccination without increasing mortality due to RV exposure prior to vaccination. However, the risk of intussusception after RV vaccination increases with infant age, so vaccinating later may not be a viable strategy [57,58]. Notably, a trial in Indonesia of the RV3-BB vaccine formulation (G3P[7], not currently endorsed by WHO) demonstrated better efficacy when the 3-dose series was administered earlier in life, starting at birth (75%) rather than at 8 weeks old (51%) [59]. This suggests that better efficacy can be achieved by vaccinating earlier in life and may circumvent the additional intussusception risk associated with RV vaccination in older infants.

Vaccine formulation other than oral exposure to live-attenuated virus is another potential alternative. For example, a recombinant, truncated VP4 protein was more immunogenic than live-attenuated formulations and was not inhibited in the presence of matAb in a mouse model [26]. However, the efficacy of nonreplicating RV vaccines needs to be further validated with challenge studies using human RV strains. Additionally, a nonreplicating vaccine formulation does not guarantee better infant vaccine response. For example, in a gnotobiotic piglet model of human RV disease, boosting an oral live-attenuated vaccine with RV-like particles resulted in suppression of effector and memory B cell responses [60]. Furthermore, there are several protein vaccines whose efficacies are affected by matAb interference, including tetanus and hepatitis B vaccines [24].

Another potential alternative to oral live-attenuated RV vaccines is a viral-vectored vaccine designed for long-term antigen expression. Continuous expression of antigen through vectored expression, administered early to release antigen for a longer period, could stimulate the infant immune system after matAbs drop to a noninterfering level [24,61]. However, gene therapy approaches are held to a higher safety standard due to the potential of vector integration into the genome [62], and further investigation is needed to determine if such an approach would be effective in the context of RV vaccination. While there are several possible approaches, further investigation is needed to determine if their ability to overcome matAb interference outweighs the risks to the infant.

## Prospects for overcoming matAb interference to infant RV vaccination

Effective RV vaccines currently exist, but efficacy of these vaccines is significantly reduced in LMICs. While many factors likely contribute to this reduction in efficacy, matAb interference is clearly associated with reduced vaccine efficacy, but further study is needed to isolate matAb interference as a contributing factor and fully establish a causal link. Mechanisms of matAb interference to orally administered RV vaccines may differ from those observed in other vaccines due to the importance of mucosal immunity and the potential for breast milk Abs to contribute to interference. Defining the mechanisms of matAb interference in this context will greatly inform alternative vaccination strategies to avoid or overcome matAb interference. Several alternative vaccination strategies have been proposed to reduce matAb interference, but these require further testing to determine the relative safety. Thus, more research into mechanisms of RV vaccine matAb interference and the safety and efficacy of alternative vaccination strategies is needed to ultimately achieve improved RV vaccine efficacy in LMICs and further reduce mortality from the leading diarrheal disease worldwide.
